# A comparison of capturing speech breathing using respiratory inductive plethysmography and electromagnetic articulography

**DOI:** 10.1371/journal.pone.0348123

**Published:** 2026-05-28

**Authors:** Tabea Thies, Philipp Buech, Anne Hermes

**Affiliations:** 1 University of Cologne, Faculty of Arts and Humanities, IfL Phonetics, Cologne, Germany; 2 University of Cologne, Faculty of Medicine and University Hospital Cologne, Department of Neurology, Cologne, Germany; 3 Laboratoire de Phonétique et Phonologie, CNRS, Université Sorbonne Nouvelle, Paris, France; Osaka University, JAPAN

## Abstract

This study introduces a novel approach to speech breathing analysis by evaluating Electromagnetic Articulography (EMA) for tracking speech breathing patterns and thus providing an alternative to the commonly used Respiratory Inductive Plethysmography (RIP). We recorded speech production data from 18 speakers (9 F, 9 M), aged between 23 and 54 years, producing sentences that differ in length and syntactic complexity. Multiple EMA sensor configurations were evaluated to determine which measures best reflect the RIP signal. For the purposes of this study, the analysis was restricted exclusively to thoracic (chest) movements for both EMA and RIP. The results demonstrate that EMA reliably captures key respiratory events in speech, including inhalation and exhalation duration as well as full breathing cycle trajectories. Horizontal EMA distance measures, particularly those derived from a sternum or middle rib cage sensor combined with a sensor on the lowest cervical vertebra, showed the highest correspondence with RIP across temporal and spatial dimensions. Distance measures of these EMA sensors exhibited strong correlations with RIP signals and reproduced comparable speech breathing patterns in the temporal and spatial domain. Vertical EMA measures were more variable, but did not compromise the overall detection of speech breathing intervals, such as inhalation and exhalation phases. In sum, these findings highlight that EMA can be an effective method for the kinematic analysis of articulatory and speech breathing data. The proposed two-sensor setup is easy to apply and provides robust speech breathing data without additional instrumentation. This approach offers a practical path for incorporating speech breathing assessment into articulatory research and has the potential to advance our understanding of speech production in linguistic and clinical contexts.

## Introduction

Breathing plays a central role in speech production, functioning as the motor that drives the entire process by providing the necessary airflow for producing speech [1,2]. Through the activation of the phonatory system and the support of the articulatory system, this airflow is transformed into speech sounds. Various conditions, such as pulmonary or neuromuscular diseases, can disrupt the neural and physiological mechanisms underlying speech breathing, leading to measurable alterations in speech production. Additionally, factors such as aging can influence breathing in general, making the study of speech breathing relevant not only for linguistic research but also for understanding how it is affected by aging and disease [3–9]. Investigating breathing patterns during speech thus provides valuable insights into both typical speech production and the physiological changes associated with health conditions.

**Fig 1:**
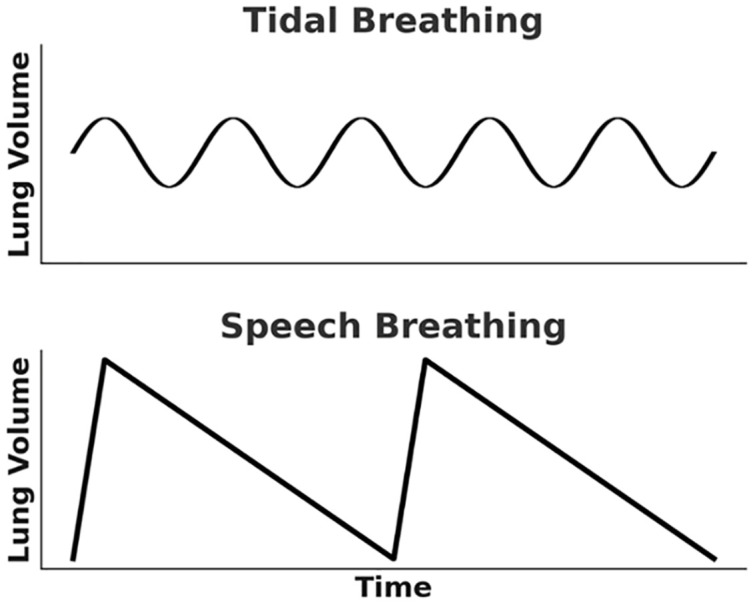
Schematic presentation of tidal breathing (top) and speech breathing (bottom).

Speech breathing has been studied over the past decades, with early investigations providing foundational insights into the mechanisms between breathing and speech production [10–13]. Unlike tidal breathing, which refers to relaxed, passive breathing at rest, characterized by relatively shallow inhalations and exhalations without conscious effort, speech breathing is an actively controlled process [14]. It involves inhaling a greater volume of air than during tidal breathing, followed by a gradual, sustained exhalation during speech. Fig 1 represents the two types: Tidal breathing displays a regular, nearly periodic oscillatory pattern, whereas speech breathing is marked by a rapid, deep inhalation followed by a prolonged exhalation, resulting in an asymmetric pattern. These two phases of speech breathing are usually described in previous studies [1,15–19].

Previous research on speech breathing has demonstrated that respiratory patterns during speech are systematically modulated by a range of linguistic, cognitive, and situational factors. For example, breathing patterns differ across modalities such as listening versus speaking [20–22], and is further influenced by physical states such as exercise [21,23], leading to an increase in inhalation rates. Linguistic factors, including sentence length and syntactic complexity, pausing behavior, speaking rate, and speech loudness, also shape the temporal and spatial characteristics of the speech breathing cycle [1,2,24–26]. In terms of speech-planning processes, speakers typically inhale at syntactic boundaries and take deeper inhalations before longer utterances, anticipating the lung volume required to complete an utterance on a single breath. Interactional contexts likewise influence respiratory patterns. During conversational turn-taking, the coordination between interlocutors can, for example, lead to breath holds early in the exhalation phase, illustrating that speakers actively regulate their breathing in response to turn-taking demands [15,20,27]. Moreover, interactions between the respiratory and the phonatory as well as articulatory systems have been described [28,29], highlighting the research potential of simultaneously capturing articulatory and breathing data in speech.

For studying speech breathing patterns, Respiratory Inductive Plethysmography (RIP) is a widely utilized method that is valued for its non-invasive nature and capacity to capture detailed respiratory dynamics [2,30–32]. Currently, simultaneous recording of articulatory and speech breathing kinematics requires the integration of two distinct systems, typically, a RIP system for speech breathing and a kinematic tracking system, such as Electromagnetic Articulography (EMA), for articulatory movements. The use of multiple systems can introduce logistical challenges, increase experimental complexity, and may impact participant comfort and the synchronization of recorded data.

In this study, we introduce a novel approach that uses EMA to capture articulatory kinematics alongside respiratory kinematics, i.e., speech breathing pattern. This dual-functionality approach aims to facilitate data collection on speech breathing in combination with speech articulation, to reduce the technical burden on researchers, and to provide a more comprehensive understanding of the coordination between speech-related subsystems. To achieve this, we provide a detailed methodological description of how speech breathing data were captured using the EMA AG 501 system, including sensor placement, signal processing, and calibration procedures necessary to ensure accurate and reliable measurements. Further, we conducted comparative analyses of RIP and EMA signals collected during the same recording session to underline that EMA indeed is a suitable system for speech breathing research. The simultaneous assessment of articulatory movements and speech breathing patterns opens new possibilities for investigating the coordination of these two speech subsystems.

### Plethysmography as standard for capturing speech breathing

To clarify how our approach differs from current practice, we briefly outline plethysmography, the predominant method for recording respiratory kinematics. The use of plethysmography is a common technology and valuable tool for respiratory data collection in research but also clinical practice and other applied settings. Several devices exist that make use of RIP, e.g., Inductotrace (Ambulatory Monitoring, Inc.) or RespitraceTM. Generally, each of these devices consists of two (elastic) bands (with insulated wires) that are positioned around the rib cage (the upper edge positioned near the level of the axilla) and the abdomen (with the upper edge approximately at the level of the umbilicus) to track thoracic and abdominal volume changes during inhalation and exhalation. The technique uses wire coils, an oscillator, and frequency demodulation electronics that track frequency shifts, converting them into digital outputs, e.g., a waveform. When a person breathes in, the rib cage and abdomen expand, which modifies the inductance of the coils [33]. With appropriate calibration, the two signals can be summed to approximate tidal volume, where the magnitude of the waveform increases with the amount of air inhaled.

The RIP bands are available in different sizes, so that they are adjustable to participants’ needs and physical conditions. The recent incarnation of the RIP, the RespTrack system, was developed by Columbi Computers AB (Sweden). It consists of one main unit, cables and adjustable inductance belts eliminating the need for different band sizes [15,34]. The advantages of these RIP systems are that they are easy to apply and feature a workflow that is manageable to learn and reliably validated. However, there are also some limitations that come along with those systems, such as that wearing the bands may affect participants’ comfort and awareness of the equipment which could further lead to alterations in breathing behavior. Another limitation is that body motions, such as arm and shoulder movements, can generate artifacts in the signal that can affect the accuracy of the data [1].

As smaller and/or wireless sensors improve comfort during breathing, other techniques such as structured light plethysmography or optoelectronic plethysmography might be favored to collect breathing patterns. Particularly, optoelectronic plethysmography is a 3D motion capture technique that provides a non-invasive assessment by placing small reflective markers on the person’s body, e.g., around the rib cage and abdomen. Several cameras around the person track how these markers move as they breathe. This method is therefore not only capable of recording breathing patterns at resting positions but also during physical activity, further offering new variables to determine speech breathing [35,36].

However, we are particularly interested in evaluating EMA as a technology that can capture not only speech articulation but also speech breathing, enabling the use of a single integrated system. Previous approaches have combined a plethysmography setup with an articulatory recording system using, e.g., EMA [24]. In that study, speech breathing data was captured with RIP, while articulatory data was collected using EMA. In the present study, we introduce the use of EMA, considered as one of the standard methods for capturing articulatory data, for tracking both articulatory and speech breathing data.

### Electromagnetic articulography as standard for tracking articulation

EMA generates a low-strength electromagnetic field by using transmitter coils within the machine. These transmitter coils are fixed in space and placed around the head. Small receiver sensors, sized approximately 3 x 3 mm, are usually attached to the articulators, such as tongue, lips, and jaw. Those sensors respond to the electromagnetic field, so that the system captures 3D movement high temporal and spatial resolution data during speaking. As studies on sign language [37], head movement [38] or eyebrow movement [39] show, all sensors’ movements that are placed in the electromagnetic field get tracked. Further information about the general practice using EMA can be found here [40,41].

In contrast to the RIP which measures changes in expansion and contraction of rib cage and abdomen translating it into volume changes, EMA directly captures the precise position, angle and motion of sensors in a 3D space. For the AG 501 from Carstens Medizinelektronik GmbH movements are captured with an accuracy of about 0.3 mm (RMS) and a sampling rate of 1250 samples/s [40,42]. Thus, while RIP only provides changes over time, EMA sensors can be analyzed in different directions, opening the path to investigate breathing patterns in more detail.

While RIP provides valuable information, it cannot capture fine-grained dynamics as well as the coordination between speech breathing and articulation. Building on these capabilities, the following section describes our methodology for using the EMA system to capture speech breathing kinematics, including sensor placement, calibration procedures, and signal processing.

## Methodology

### Participants

**Fig 2:**
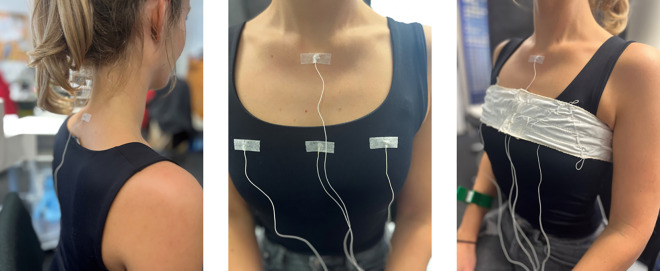
Placement of EMA sensors attached with tape on the participant and positioning of the RIP belt.

Eighteen native German speaking participants (9 males, 9 females) were enrolled in the study between 7 and 9 November 2023. The age ranged from 23 to 54 years with a mean age of 33 years (SD = 9 years). To prioritize the technical evaluation of the experimental setup, we intentionally avoided restrictive inclusion or exclusion criteria in the recruitment of participants. Consequently, we did not exclude participants based on factors such as smoking status, respiratory conditions, or body mass index (BMI), as our primary objective was to validate the system across a diverse range of speaker ages and body types. The study was approved by the ethics committee of the “Deutsche Gesellschaft für Sprachwissenschaft” (2020-04-200327). Written informed consent was obtained from all participants involved in the study. Further, written informed consent was obtained from the individual in Fig 2 for the publication of these photographs.

### Setup description

To ensure reliable signal quality from EMA sensors, participants were advised beforehand to wear tight-fitting shirts during the recording session to prevent artifacts from too loose clothing or folds of clothes. Kinematic data for speech breathing were simultaneously collected using EMA (AG 501, Carstens Medizinelektronik GmbH) at a sampling rate of 1250 Hz and RIP (Inductotrace®, Ambulatory Monitoring, Inc.) at a sampling rate of 44.1 kHz. Respiratory data were recorded exclusively from thoracic movement. Given that participants remained seated throughout the session, abdominal contributions to speech breathing were expected to be minimal.

#### EMA signals.

For tracking speech breathing data with EMA, sensors were attached and secured with tape (Fig 2, left and middle panel). A sensor on the lowest vertebra of the cervical spine directly on the skin served as the reference sensor (referred to as R, Fig 4).

**Fig 3:**
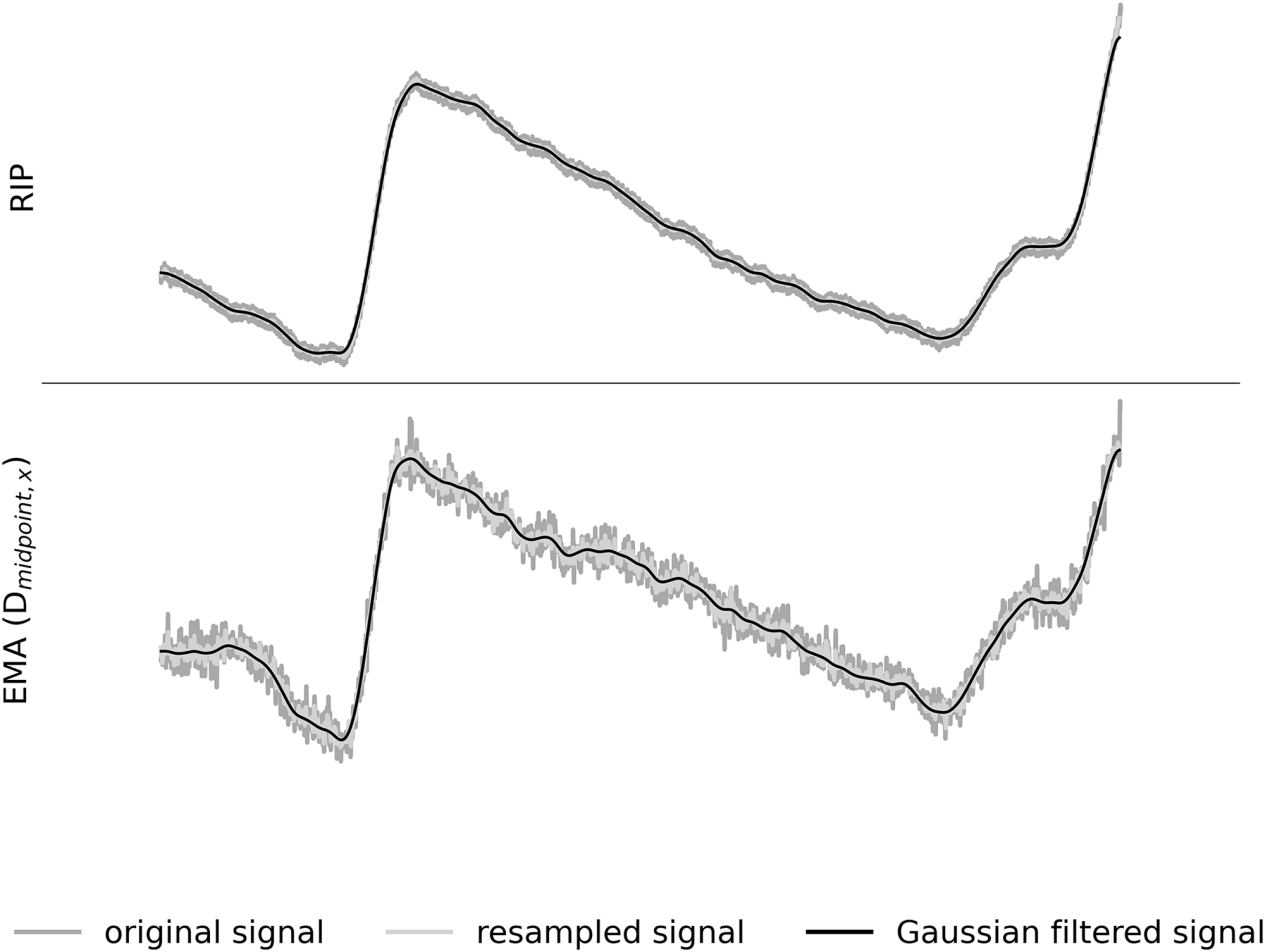
Exemplified effects of downsampling and filtering on the two signals (speaker P15, long simple sentence), showing the RIP signal (top) and the EMA signal (bottom). EMA signal is represented by a vertical distance (D_midpoint,x_) between the calculated midpoint (located between the left and right chest sensors) and the reference sensor. Dark grey lines represent the original signal, light grey lines show the resampled signals and the black lines are the Gaussian filtered signals.

**Fig 4:**
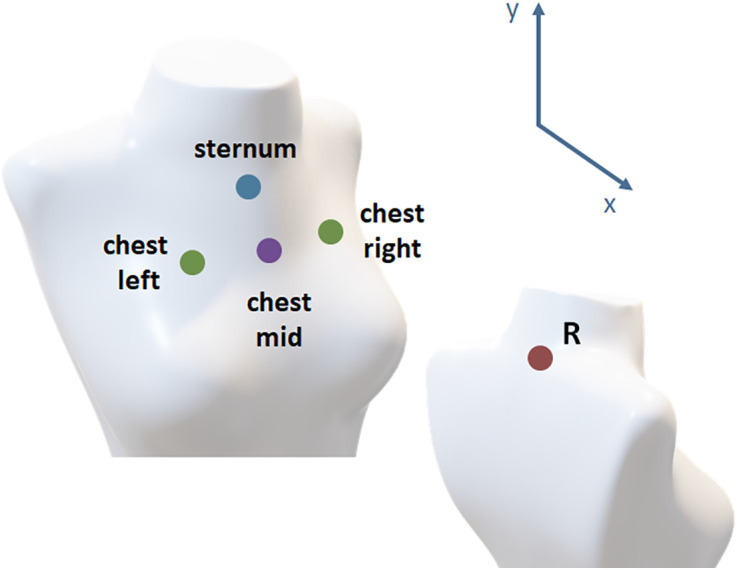
Schematic view of EMA sensors placement on the participants’ upper body part to capture breathing kinematics calculating distances (D) between motion sensors and the reference sensor (R).

Additional sensors were placed on the sternum (on the skin) and three locations on the rib cage to monitor (speech) breathing kinematics. To track thorax movements, sensors were positioned at axilla level on the chest (on clothes), with one sensor in the center and two aligned with the level of each papilla (Fig 4, chest left, chest mid, chest right). After the EMA sensors were positioned, a RIP band (only the upper band for thoracic movement) was placed around the participant’s rib cage (Fig 2, right panel). As body sizes differed between the participants, three different RIP band sizes were used (7 x small, 5 x medium, and 6 x regular). Participants were seated on a chair below the EMA system and in front of a screen that presented the different tasks.

#### RIP signals.

To ensure accurate measurement of breathing signals, the RIP system was calibrated prior to each experimental session. The calibration procedure included selecting an appropriately sized RIP band to ensure a secure fit around the participant’s chest. This placement remained unchanged throughout the recording session to maintain data consistency. The red calibration button on the device was pressed to activate the calibration mode. Signal baselines were adjusted manually via the upper channel-specific controls so that the displayed signal fluctuated around zero, ensuring a balanced baseline. To then normalize the RIP signal to the individual participant’s respiratory range, a maximum inhalation and exhalation procedure was conducted. Participants were seated and instructed to inhale and exhale as deeply as possible to determine the minimum and maximum respiratory volumes. The duration of inhalation/exhalation phases was not critical as only the full range of movement (i.e., amplitude) was relevant. This process was repeated three times with sufficient pauses between trials to avoid dizziness or hyperventilation. Calibration quality was monitored through initial zero-setting, ongoing plausibility checks, and continuous inspection of the RIP belt for potential slippage. No repositioning or adjustment of the belt was required for any participant during the recording sessions. Signal values were deemed acceptable if, following the zero-setting procedure, they fluctuated symmetrically around the baseline and exhibited waveform patterns consistent with tidal breathing during non-speech intervals.

Throughout data acquisition, calibration quality as well as the position of the RIP band was continuously monitored. Specifically, we checked whether the real-time display of breathing motion which was displayed in EDWin tool, a software designed for processing, visualizing, and analyzing pressure measurements, corresponded in a plausible way with the breathing pattern of the participant, and whether the signal values remained within a possible range.

### Tasks

Acoustic and kinematic data (of the RIP and the EMA) were recorded during several tasks, such as i) maximal inhalation and exhalation, ii) tidal breathing, iii) maximum phonation of vowel /a/, iv) sentence production, and v) reading text.

For i), participants were asked to inhale and exhale as deeply as possible, while duration and speed did not matter. The task was completed three times. For ii), participants were having a short break to relax; to ensure that instructions or task awareness did not influence their typical tidal breathing patterns. Tidal breathing was recorded for 30–45 s [43]. For iii), they were asked to breathe in deeply and produce the vowel [a] for as long as possible until they ran out of air. This task was also completed three times. For iv), we used some of the sentences that were used before by Fuchs et al. [2] which differ in length and complexity (see target sentences 1–4). Each sentence was produced twice. For v), participants read the text “Nordwind und Sonne (The North Wind and the Sun)”.

Two tasks, i.e., sustained vowel phonation and text reading, were produced in habitual and loud speaking style. For loud speech, participants were asked to keep a constant level of 80 dB. The tracking of speech loudness was done via a sound level meter that was positioned 1.25 m away from the participants.

In a preceding analysis on sustained vowel productions, similar speech breathing patterns between RIP and EMA in sustained vowel productions have already been observed [44], showing that the temporal parameters, i.e., inhalation and exhalation phases, between EMA and RIP in habitual and loud speech were similar. While particularly for exhalation phases no significant differences were found, inhalation phases were slightly shorter when captured with EMA sensors. In addition, comparable movement contours across the entire breathing cycle were reported. Building on the first indications that EMA is capable of tracking similar patterns to RIP [44], we provide a full comparison of RIP and EMA in speech during sentence productions differing in length and complexity (taken from [2]). We only used a subset of the sentence repertoire, using the following target sentences per condition:

1) **Short simple:** Sonja Wunderlich besuchte die Komische Oper. (*Engl.: Sonja Wunderlich visited the Komische Oper.*)
2) **Short complex:** Sonja Wunderlich, die Tanz liebt, besuchte die Oper. (*Engl.: Sonja Wunderlich, who loves dance, visited the opera.*)
3) **Long simple:** Sonja Wunderlich bestaunte in einer warmen Sommernacht im Monat August die Oper. (*Engl.: Sonja Wunderlich admired the opera on a warm summer night in the month of August.*)
4) **Long complex:** Sonja Wunderlich sagte einem Freund, der uns morgens anrief, sie begeistert sich für Oper. (*Engl.: Sonja Wunderlich told a friend, who called us in the morning, that she is enthusiastic about opera.*)

Each of these sentences was preceded by the same introductory trigger sentence: “In der Wochenendausgabe der Berliner Zeitung von Anfang Dezember 1996 stand geschrieben:” (*Engl.: The weekend edition of the Berliner Zeitung from the beginning of December 1996 contained the following:*). This trigger sentence was intended to control participants’ inhalation, ensuring that a new breathing cycle began before the production of the target sentence. Participants were instructed to read the sentences as naturally as possible, with no further constraints regarding tempo or loudness.

### Postprocessing, data analysis & measures

#### Signal processing.

The RIP and the EMA signals were recorded with separate synchronized audio tracks. Audio was captured using two identical head-mounted microphones (AKG C544 L), each connected to its respective recording system. To ensure consistent input for synchronization, both microphones were placed adjacent to one another on the arm of the EMA, positioned in close proximity to the participant. Notably, participants did not wear the headsets, as the study’s objective did not require detailed acoustic analysis beyond signal alignment. All recordings were conducted in a controlled environment with low ambient noise to ensure the clarity of the acoustic synchronization impulses. The synchronization of the RIP and the EMA signal was done via the acoustic signal based on an acoustic impulse produced with a clapperboard at the beginning of the recording. Afterwards, the acoustic onset and offset of each speaker’s production were segmented using Praat [45]. The RIP signal was extracted from the second channel of the RIP recording as it contained the breathing data. The raw kinematic EMA trajectories of the vertebra, sternum and the rib cage sensors, that were not corrected for head-movements (as it is usually done with EMA data), were extracted using the data extraction routine from the ADA tool [46]. In addition, each kinematic trajectory was normalized similar to the RIP recording by dividing the amplitude of each sample by the maximum of the absolute signal value. Both the RIP and the EMA data were downsampled to 100 Hz and a one-dimensional Gaussian kernel filter was applied, as implemented in SciPy v1.16.1 [47], with sigma set to 5. Fig 3 illustrates the effect of the downsampling and the filtering on the data. The original raw signals are represented in dark grey and the processed signals are represented in light grey (resampled signal) and black (Gaussian filtered signal) lines. As can be seen in Fig 3, the raw kinematic signal exhibits more noise compared to the raw RIP signal, although overall the kinematic pattern is clearly observable.

#### EMA distance measures.

By using all four EMA sensors that were positioned on each participant’s chest plus one EMA sensor serving as a reference, three distance measures were calculated between EMA motion sensors and the EMA reference sensor (Fig 4).

Each distance D was calculated in two dimensions, x (front-back) and y (low-high):


$$\begin{array}{c} D_{chestmid}\;=\;{EMA}_{chestmid}-\;{EMA}_{ref} \end{array}$$ (1)

$$\begin{array}{c} D_{\text{midpoint}}\;=\left(\frac{{EMA}_{left}+{EMA}_{right}}{\text{2}}\right)-{EMA}_{ref} \end{array}$$ (2)

$$\begin{array}{c} D_{sternum}\;=\;{EMA}_{sternum}\;-\;{EMA}_{ref} \end{array}$$ (3)

The first distance measure, D_chestmid_, is assigned between the chest’s middle sensor and the reference sensor. The second distance measure, D_midpoint_, was taken between a calculated midpoint between the left sensor and right sensor on the chest and the reference sensor. Finally, the third distance measure, D_sternum_, was taken between the sternum and the reference sensor. In this analysis, we focus on single dimensions to align with the methodological approach, understand the data and to be able to compare it with the RIP data.

#### Variables of interest.

**Fig 5:**
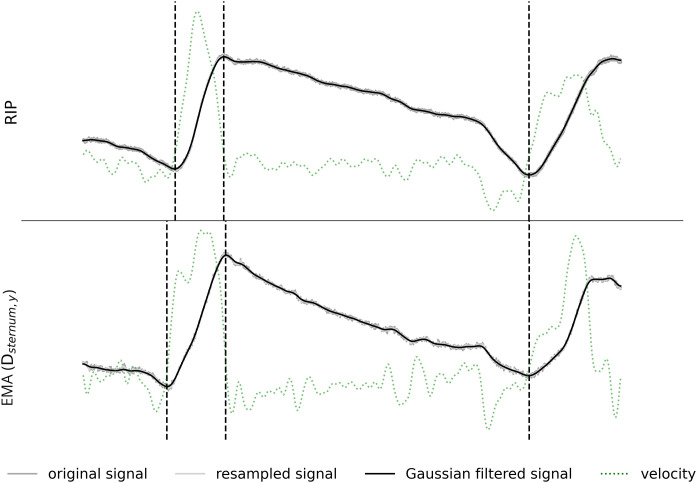
Example of automatic landmark detection in the EMA and RIP signal respectively (speaker P13, long simple sentence). **RIP signal (top) and EMA distance measure signal (bottom, D**_**sternum,y**_ **). Landmarks are highlighted by dashed vertical lines in the following order: inhalation onset, inhalation offset/ exhalation onset, and exhalation offset.**

For all speech breathing cycles, the three most commonly used landmarks were defined: i) inhalation onset, ii) inhalation offset/exhalation onset, and iii) exhalation offset. This was done automatically by the following steps as can be seen in Fig 5: First, the maximum of the velocity profile (green line) of the filtered RIP and the EMA distance measure was located within a window of two seconds centered at the acoustic onset of the word “Sonja“. Then, the inhalation onset was identified by the first zero crossing to the left of the velocity peak (first vertical line in Fig 5). The inhalation offset/exhalation onset was identified by the first maximum to the right of the velocity peak (second vertical line in Fig 5). Finally, the exhalation offset was set at the local minimum in a window from the velocity peak and the acoustic offset of the utterance with additional four seconds (third vertical line in Fig 5). The automatically defined landmarks were inspected afterwards and manually corrected if necessary.

**Fig 6:**
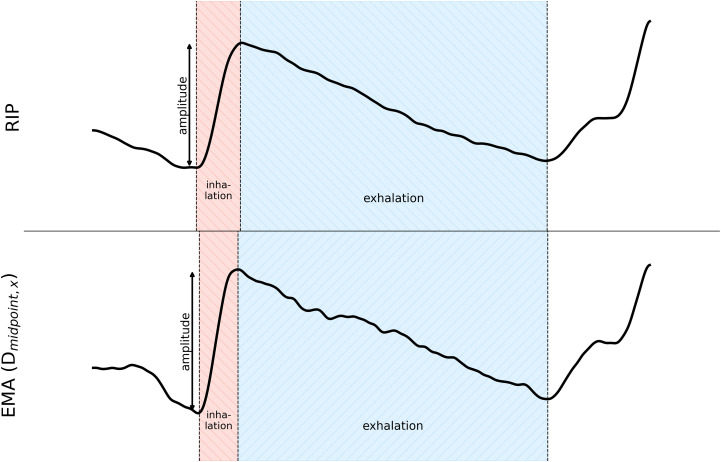
Example of the variables of interest (speaker P15, long simple sentence) with RIP signal (top) and EMA distance measure D_midpoint,x_ (bottom). The inhalation amplitude is marked by an arrow; the inhalation duration is indicated by a red rectangle and the exhalation duration by a blue rectangle.

Based on these landmarks, we calculated three temporal parameters: i) inhalation duration, ii) exhalation duration, and iii) time lag Δ_*Sonja*_. As Fig 6 illustrates, the inhalation duration is the time interval from inhalation onset (minima) to inhalation offset (maxima), while the exhalation duration is the time interval from inhalation offset/exhalation onset (maxima) to the exhalation offset (minima); which is often also the inhalation onset of the preceding breathing cycle. Both intervals add up to one breathing cycle. In addition, one spatial parameter, the inhalation amplitude, was measured from the inhalation onset to the inhalation offset.

To capture the temporal alignment between the acoustic level and kinematic level, the interval between the acoustic onset of the word “Sonja” and the inhalation was calculated and assigned as “Δ_Sonja_” for both the RIP and the EMA signal.

Note that the units of the RIP and the EMA signals differ: While the former are expressed in normalized sample values, the latter are recorded in millimeters. To overcome these differences for the spatial measure, the sample values from the inhalation onset to the exhalation offset were z-scored and the inhalation amplitude was subsequently extracted from the inhalation onset to the inhalation offset from the standardized cycles and are thus expressed in standard deviations.

Finally, the RIP and EMA trajectories were extracted to compare the signals’ dynamics over time. A window reaching from the inhalation onset −1.5 seconds to the exhalation offset +1.5 seconds based on the RIP annotations was used for the time series extraction.

#### Statistics.

144 utterances (4 target sentences x 2 repetitions x 18 participants) were recorded in this sentence production study. Two speakers were removed entirely from the analysis as they did not produce the expected breathing pattern, i.e., inhalation between the trigger sentence and the target sentences. Of the remaining 128 utterances, 14 sentences were also excluded for the same reason, corresponding to 10.9% of the remaining data. For all excluded utterances in which no inhalation before the target sentence was observed in the RIP signal, no inhalation was observable in the EMA signal either. In total, 114 sentences were included in the analysis (28 x short simple, 27 x short complex, 30 x long simple, 29 x long complex) for the remaining 16 speakers. The summary statistics are expressed in medians and interquartile ranges (IQR) to provide a better overview of the respective distributions. In addition, we computed the Jensen-Shannon distances of the probability densities of the EMA distance measures with the RIP data as a measurement of similarity between their distributions. Values close to 0 indicate higher similarity, whereas values close to 1 indicate greater dissimilarity. The datasets and analysis scripts supporting the findings of this study are archived on the Open Science Framework (OSF) within the project “Comparison of Respiratory Inductive Plethysmography and Electromagnetic Articulography.” These materials are publicly accessible at https://osf.io/wt543/ or via https://doi.org/10.17605/OSF.IO/WT543.

To compare parameters across signals between EMA and RIP, we analyzed the correlations of one-dimensional parameters, namely inhalation duration, exhalation duration, time lag Δ_Sonja_, and inhalation amplitude. This approach follows [48] who investigated associations between nasalance measurements obtained from two different methods (earbud nasalance scores and nasometer nasalance scores). For our analysis, we used Bayesian multivariate normal models to examine pairwise comparisons of the RIP signal with the EMA distance measures per sentence type. Prior to modeling, all parameter values were standardized. For the models mean, we used a normal prior centered at 0 and with a standard deviation of 1.5. The covariance matrix of the multivariate normal distribution was modeled using Lewandowski–Kurowicka–Joe (LKJ) Cholesky priors with *η* set to 1, which avoids bias toward higher or lower correlations. Standard deviations were assigned Exponential priors with *λ* = 1. Models were implemented in PyMC v.5.22.0 [49] and run with four Markov Chain Monte Carlo (MCMC) chains, each consisting of 8000 tuning samples and 8000 draws, resulting in a total of 32,000 posterior samples. For the one-dimensional analyses, we report the posterior mean (ρ) along with the lower and upper boundaries of the 95% highest density interval (HDI). Decision-making was based on whether the HDI included the null value. Additionally, we report the posterior probability above zero (Pr($\hat{p}$ > 0)) as a measure of effect size.

The signal dynamics were analyzed using Gaussian Process (GP) models. For these dynamic analyses, the trajectories were extracted at 50 equally-distanced steps. Separate models were run for each sentence type. Within each model, one GP was fitted for each dimension as common level effects and a by-speaker intercept was used as group level effect. The model structure was as follows: time series ~ 0 + GP_signal_ + (1|Speaker).

Exponential priors were used for the GPs, with *λ* set to 2 for the amplitude as the trajectory amplitudes are normalized and *λ* set to 3 for the length scale since the extracted trajectories of the breathing cycles have a clear rise at their beginning. For the GP analysis, the Hilbert Space Gaussian Process approximation was used as described by [50] and as implemented in Bambi v. 0.15.0 [51] with the number of basis vectors set to 50 and the extension factor to 2. A HalfNormal prior with σ = 1 was used for the variance, while default priors were used for the remaining coefficients. Each model was run with four MCMC chains and 4 000 samples for tuning and 4 000 draws; 16 000 samples were thus used for inference. We report the estimates for the RIP and the EMA distance trajectories as well as their difference. Similar to the parametric analyses, we used the difference between the two signals. Besides this dynamic analysis, we calculated also the cross-correlations from the entire trajectories to parametrize the overall similarity of the shapes of the speech breathing cycles displayed by RIP and the EMA distances.

## Results: Speech breathing patterns in RIP and EMA

### (Non-)Interference of EMA with RIP and EMA without RIP

**Fig 7:**
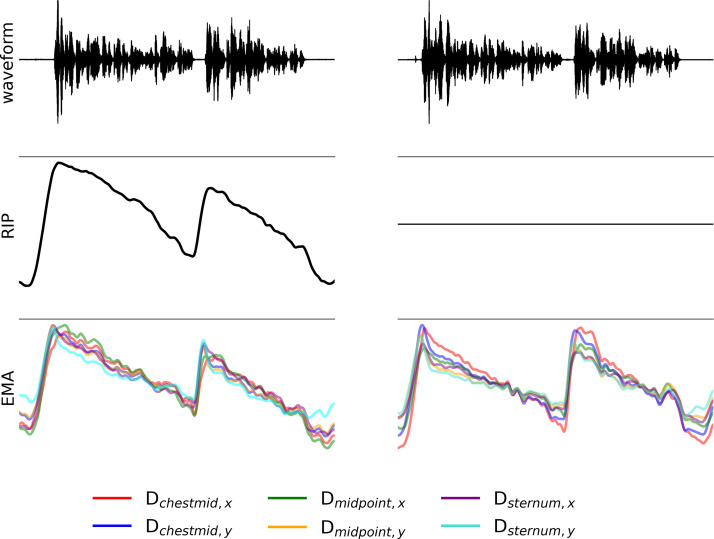
Signal comparison of two breathing cycles of the same utterance and the same speaker (speaker P8, trigger sentence followed by long simple sentence).Left: RIP and EMA, right: EMA only. Signals are downsampled to 100 Hz.

In a first step, we aimed to test whether the RIP belt influences the EMA signal as in our setup the RIP belt was positioned over the EMA sensors. To examine whether the belt interfered with the EMA signals, exemplarily recordings were conducted for one speaker in two conditions in this consecutive order: i) with both EMA and RIP, and ii) EMA alone (without RIP). Trajectories from both recordings were compared visually as a validation step to ensure there was no interference between the RIP belt and EMA sensors (Fig 7).

The left panel of Fig 7 displays RIP and EMA from the first recording, while the right one shows EMA only from the second recording. Each example shows the production of the trigger sentence followed by the long simple sentence. From top to bottom, the visualization includes acoustics, RIP (which is present on the left but a flatline on the right), followed by different EMA signals. For the EMA signal, the superimposed calculated EMA distances (D_midpoint_, D_sternum_, D_chestmid_) are presented. The example demonstrates the same sentences, and when focusing solely on the EMA signals, the speech breathing patterns resemble across different distance measures and recordings. This underlines that that RIP does not interfere with EMA.

### Temporal measures

**Fig 8:**
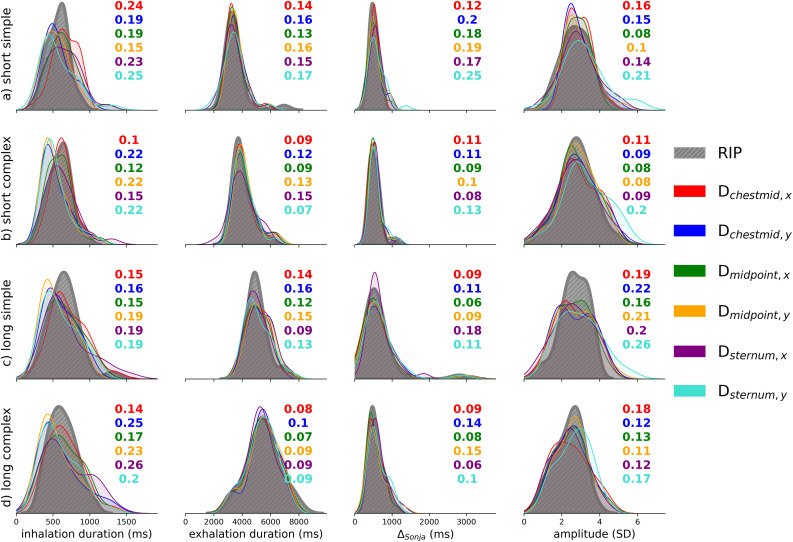
Jensen–Shannon distances as a similarity measure between the distributions of the EMA distances (color-coded) and RIP (in grey). Mean values close to 0 indicate higher similarity, whereas values close to 1 indicate greater dissimilarity.

The analysis of the temporal variables refers to the three different parameters under investigation, i.e., inhalation duration, exhalation duration, and Δ_*Sonja*_. The presentation of the results will follow this order for each variable to assess comparability between RIP and EMA distance measures: i) medians and with the IQR per dimension and sentence type (Table 1, Table 3, Table 5), b) correlation coefficients between RIP signal and each EMA distance measure per sentence type (Table 2, Table 4, Table 6), and c) Jensen-Shannon distances of the probability densities as a measurement of similarity between the EMA-RIP dimensions (Fig 8). Speaker-specific values can be found in the OSF project.

**Table 1:** Speech inhalation durations. Values are presented as the median with the IQRs in brackets by dimension and sentence type.

	sentence type			
dimension	short simple	short complex	long simple	long complex
RIP	584 (474 - 653)	633 (492 - 687)	641 (530 - 740)	593 (515 - 753)
D_chestmid,x_	657 (553 - 835)	634 (538 - 694)	615 (558 - 875)	626 (494 - 799)
D_chestmid,y_	530 (445 - 653)	458 (396 - 636)	553 (431 - 752)	493 (392 - 729)
D_midpoint,x_	610 (481 - 760)	599 (471 - 673)	638 (474 - 797)	599 (484 - 864)
D_midpoint,y_	519 (423 - 668)	466 (402 - 638)	507 (410 - 726)	465 (387 - 722)
D_sternum,x_	621 (523 - 791)	594 (491 - 716)	579 (489 - 831)	602 (449 - 907)
D_sternum,y_	480 (413 - 747)	481 (426 - 588)	552 (391 - 806)	497 (392 - 786)

**Table 2:** Correlation coefficients for the inhalation duration. Means as well as the lower and upper boundaries of 95% of the posterior by EMA dimension and sentence type.

	sentence type											
	short simple			short complex			long simple			long complex		
dimension	$\hat{𝐩}$	95% HDI	Pr($\hat{𝐩}$ >0)	$\hat{𝐩}$	95% HDI	Pr($\hat{𝐩}$ >0)	$\hat{𝐩}$	95% HDI	Pr($\hat{𝐩}$ >0)	$\hat{𝐩}$	95% HDI	Pr($\hat{𝐩}$ >0)
D_chestmid,x_	.29	[-.05, .62]	.94	.64	[.41, .84]	1.0	.75	[.58, .89]	1.0	.78	[.64, .91]	1.0
D_chestmid,y_	.2	[-.14, .54]	.87	.61	[.36, .82]	1.0	.63	[.41, .83]	1.0	.66	[.44, .85]	1.0
D_midpoint,x_	.37	[.05, .67]	.98	.67	[.45, .86]	1.0	.57	[.32, .79]	1.0	.81	[.68, .92]	1.0
D_midpoint,y_	.19	[-.15, .53]	.86	.62	[.37, .82]	1.0	.69	[.5, .86]	1.0	.6	[.36, .81]	1.0
D_sternum,x_	.2	[-.14, .54]	.87	.56	[.3, .8]	1.0	.7	[.52, .86]	1.0	.73	[.55, .88]	1.0
D_sternum,y_	.3	[-.02, .62]	.96	.58	[.32, .81]	1.0	.65	[.43, .83]	1.0	.65	[.43, .83]	1.0

#### Inhalation duration.

When comparing inhalation durations, derived from RIP and EMA distance measures, particularly D_chestmid,x_ and D_midpoint,x_ showed the strongest agreement with RIP. Across all sentence types, RIP and EMA signals displayed a strong overlap. For example, in short complex sentences, inhalation duration was 633 ms for RIP and 634 ms for D_chestmid,x_, while in long simple sentences it was 641 ms in RIP and 638 ms in D_midpoint,x_ = 638 ms, each with tight IQR. In contrast, D_chestmid,y_, D_midpoint,y_, and D_sternum,y_ showed lower medians and broader IQR compared to RIP. For instance, in long complex sentences, RIP = 593 ms whereas D_chestmid,y_ = 493 ms, a difference of about 100 ms. Despite these measurement differences, both RIP and EMA distance measures showed adjustments due to sentence type: Independent of the underlying signal (EMA or RIP), the inhalation durations were overall longer in long sentences compared to short sentences, but mainly shorter in complex sentences than in simple sentences.

The correlation estimates in Table 2 show in general a high positive correlation of the inhalation durations measured from the EMA distances with those extracted from the RIP signal. The EMA distance measures with the highest correlation coefficients are: D_chestmid,x_, and D_midpoint,x_. High correlations (above .5) were found in short complex, long simple and long complex sentences. In short simple sentences, the posterior estimates of the correlations for almost all EMA distances reveal low correlations, except for D_midpoint,x_ where the posterior was $\hat{p}$ = .37 [.5, .67]. More detailed, for short complex sentences, correlation coefficients ranged from $\hat{p}$ = .56 [.30, .80] in D_sternum,x_ to $\hat{p}$ = .67 [.45, .86] in D_midpoint,x_. In long simple sentences, values ranged from $\hat{p}$ = .57 [.32, .79] in D_midpoint,x_ to $\hat{p}$ = .75 [.58, .89] in D_chestmid,x_. For long complex sentences, the lowest correlation of $\hat{p}$ = .60 [.36, .81] was observed in D_midpoint,y_, while the highest was found in D_midpoint,x_ with $\hat{p}$ = .81 [.68, .92].

As it can be seen in Fig 8, the Jensen–Shannon distance for inhalation duration remain relatively low (approximately 0.10–0.26) across all sentence types, indicating that the EMA-based distributions are broadly similar to the RIP reference.

#### Exhalation duration.

Following the analysis of inhalation duration, we proceed to analyze the exhalation phase of the speech breathing cycles. When comparing exhalation durations obtained from RIP and the EMA distance measures, D_chestmid,x_ and D_midpoint,x_ showed again the strongest overlap with RIP. Median differences rarely exceeded 60 ms, and IQRs overlapped substantially across all sentence types. For example, in long complex sentences, RIP = 5515 ms and D_midpoint,x_ = 5551 ms, indicating almost identical estimates. On the contrary, vertical EMA distance measures such as D_chestmid,y_, D_midpoint,y_, D_sternum,y_ produced slightly longer and more variable medians. For instance, in short simple sentences, RIP = 3260 ms while D_chestmid,y_ = 3475 ms. Across both RIP and EMA distance measures, exhalation duration changed systematically with sentence length and syntactic complexity. As shown in Table 3, exhalation durations were overall longer in long sentences compared to short sentences and tended to increase further in complex sentences, relative to simple sentences across both RIP and EMA signals.

**Table 3:** Speech exhalation durations. Values are presented as the median with the IQRs in brackets by dimension and sentence type.

	sentence type			
dimension	short simple	short complex	long simple	long complex
RIP	3260 (3106 - 3615)	3820 (3628 - 4290)	4921 (4663 - 5291)	5515 (4870 - 5983)
D_chestmid,x_	3291 (3119 - 3800)	3848 (3686 - 4230)	4997 (4655 - 5771)	5541 (4898 - 6043)
D_chestmid,y_	3475 (3256 - 3846)	3972 (3668 - 4447)	5113 (4839 - 5980)	5554 (5083 - 6177)
D_midpoint,x_	3333 (3170 - 3577)	3852 (3646 - 4442)	5036 (4533 - 5738)	5551 (4854 - 5980)
D_midpoint,y_	3468 (3283 - 3786)	3998 (3767 - 4552)	5157 (4786 - 5969)	5557 (5104 - 6292)
D_sternum,x_	3256 (3000 - 3649)	3943 (3411 - 4499)	5021 (4497 - 5443)	5286 (4886 - 5841)
D_sternum,y_	3367 (3019 - 3699)	3945 (3612 - 4391)	4905 (4388 - 5763)	5474 (4932 - 6055)

As opposed to the inhalation durations, all exhalation durations show a high positive correlation across EMA distances and sentence types with the RIP signal (Table 4). Within short simple sentences, all EMA distances correlated positively with RIP, with coefficients ranging from $\hat{p}$ = .59 [.33, .81] for D_sternum,y_ to $\hat{p}$ = .83 [.70, .93] for _Dchestmid,x_. For short complex sentences, correlations were overall stronger, ranging from $\hat{p}$ = .86 [.75, .95] in D_sternum,y_ to $\hat{p}$ = .92 [.86, .97] in D_chestmid,y_. In long simple sentences, however, correlations were weaker than in the other sentence types, with the lowest coefficient found for D_chestmid,y_ ($\hat{p}$ = .50 [.23, .76]) and the highest for D_midpoint,x_ ($\hat{p}$ = .56 [.31, .78]); nevertheless, the posterior means were closely clustered between .50 and .56. For long complex sentences, correlations were high, with values ranging from $\hat{p}$ = .69 [.49, .87] in D_chestmid,y_ to $\hat{p}$ = .75 in several EMA distances, such as D_chestmid,x_ [.58, .90], D_midpoint,x_ [.59, .90], D_midpoint,y_ [.58, .89], and D_sternum,x_ [.59, .90].

**Table 4:** Correlation coefficients for the exhalation duration. Means as well as the lower and upper boundaries of 95% of the posterior by EMA dimension and sentence type.

	sentence type											
	short simple			short complex			long simple			long complex		
dimension	$\hat{𝐩}$	95% HDI	Pr($\hat{𝐩}$ >0)	$\hat{𝐩}$	95% HDI	Pr($\hat{𝐩}$ >0)	$\hat{𝐩}$	95% HDI	Pr($\hat{𝐩}$ >0)	$\hat{𝐩}$	95% HDI	Pr($\hat{𝐩}$ >0)
D_chestmid,x_	.83	[.7, .93]	1.0	.89	[.81, .96]	1.0	.52	[.25, .76]	1.0	.75	[.58, .9]	1.0
D_chestmid,y_	.79	[.63, .92]	1.0	.92	[.86, .97]	1.0	.5	[.23, .76]	1.0	.69	[.49, .87]	1.0
D_midpoint,x_	.8	[.66, .92]	1.0	.91	[.85, .97]	1.0	.56	[.31, .78]	1.0	.75	[.59, .9]	1.0
D_midpoint,y_	.78	[.62, .91]	1.0	.91	[.84, .97]	1.0	.52	[.26, .76]	1.0	.75	[.58, .89]	1.0
D_sternum,x_	.72	[.54, .88]	1.0	.88	[.79, .96]	1.0	.53	[.26, .76]	1.0	.75	[.59, .9]	1.0
D_sternum,y_	.59	[.33, .81]	1.0	.86	[.75, .95]	1.0	.52	[.26, .77]	1.0	.73	[.55, .89]	1.0

Fig 8 depicts that exhalation duration is the parameter for which EMA distance measures most closely align with the RIP across all sentence types, as the Jensen–Shannon distances are relatively low (approximately 0.07–0.17) and the density distributions mostly overlap.

#### Time lag between acoustics and speech breathing onset: Δ_Sonja_.

The measure Δ_Sonja_ refers to the difference (time lag) between the inhalation onset of the speech breathing cycle and the acoustic onset of each sentence. Comparison of the median Δ_Sonja_ values across RIP signal with EMA distance measures revealed that D_chestmid,x_ and D_midpoint,x_ closely tracked RIP. Median differences across sentence types were generally within 15–25 ms, and IQRs showed extensive overlap. For example, for short complex sentences, acoustic onset occurred after a 520 ms time lag in RIP and 510 ms in D_midpoint,x_, with nearly identical IQR limits. Again, EMA distance measures in the vertical y-dimension demonstrated with slightly different time lags and broader IQRs relative to RIP. Δ_Sonja_ time lag values remained relatively stable across sentence types, averaging around 500 ms to 540 ms, with only minor increases for long or complex utterances (Table 5).

**Table 5:** Time lag between inhalation onset and acoustics, i.e., Δ_Sonja_. Values are presented as the median with the IQRs in brackets by dimension and sentence type.

	sentence type			
dimension	short simple	short complex	long simple	long complex
RIP	495 (430 - 592)	520 (441 - 606)	532 (438 - 670)	525 (442 - 658)
D_chestmid,x_	509 (405 - 582)	516 (404 - 559)	487 (393 - 617)	486 (387 - 693)
D_chestmid,y_	511 (423 - 590)	519 (440 - 583)	496 (423 - 778)	514 (412 - 750)
D_midpoint,x_	495 (414 - 588)	510 (416 - 567)	495 (405 - 663)	487 (392 - 689)
D_midpoint,y_	503 (433 - 600)	531 (444 - 592)	498 (436 - 729)	511 (431 - 717)
D_sternum,x_	552 (463 - 656)	552 (447 - 596)	577 (480 - 640)	576 (455 - 692)
D_sternum,y_	537 (437 - 759)	533 (479 - 608)	551 (462 - 771)	546 (451 - 817)

As presented in Table 6, Δ_Sonja_ time lags show a high positive correlation across nearly all EMA distance measures with the RIP signal (Table 4). The estimates are highest for short complex, long simple and long complex sentences. Short simple sentences had the lowest, but likewise high positive correlations, ranging from $\hat{p}$ = .61 [.37, .82] in D_sternum,x_ to $\hat{p}$ = .74 [.56, .89] in D_chestmid,x_. One exception is the relationship of the D_sternum,y_ distance with the RIP. It still has a moderate positive correlation but not as high as the others and therefore falls a bit out of the pattern. In short complex sentences, the correlation estimates were quite similar for all EMA distances ranging from $\hat{p}$ =.88 [.79, .96] for D_sternum,y_ to $\hat{p}$ = .92 [.85, .97] in D_chestmid,y_ and D_sternum,x_. The EMA distances in long simple sentences had an overall high correlation with the Δ_Sonja_, ranging from $\hat{p}$ = .87 [.78, .95] in D_sternum,x_ to $\hat{p}$ = .99 [.97, .99] in D_chestmid,x_. Finally, correlation estimates taken from long complex sentences were high and positive, with D_chestmid,x_ having the lowest correlation ($\hat{p}$ =.80 [.66, .92]) and D_midpoint,x_ ($\hat{p}$ =.96 [.93, .99]) exhibiting the highest correlation.

**Table 6:** Correlation coefficients for the Δ_Sonja_ lag. Means as well as the lower and upper boundaries of 95% of the posterior by EMA dimension and sentence type.

	sentence type											
	short simple			short complex			long simple			long complex		
dimension	$\hat{𝐩}$	95% HDI	Pr($\hat{𝐩}$ >0)	$\hat{𝐩}$	95% HDI	Pr($\hat{𝐩}$ >0)	$\hat{𝐩}$	95% HDI	Pr($\hat{𝐩}$ >0)	$\hat{𝐩}$	95% HDI	Pr($\hat{𝐩}$ >0)
D_chestmid,x_	.74	[.56, .89]	1.0	.89	[.81, .96]	1.0	.99	[.97, .99]	1.0	.95	[.91, .98]	1.0
D_chestmid,y_	.65	[.42, .85]	1.0	.92	[.85, .97]	1.0	.95	[.91, .98]	1.0	.8	[.66, .92]	1.0
D_midpoint,x_	.65	[.43, .85]	1.0	.91	[.84, .97]	1.0	.98	[.97, .99]	1.0	.96	[.93, .99]	1.0
D_midpoint,y_	.67	[.45, .85]	1.0	.91	[.83, .97]	1.0	.96	[.93, .98]	1.0	.81	[.67, .92]	1.0
D_sternum,x_	.61	[.37, .82]	1.0	.92	[.85, .97]	1.0	.87	[.78, .95]	1.0	.91	[.84, .97]	1.0
D_sternum,y_	.48	[.2, .74]	1.0	.88	[.79, .96]	1.0	.93	[.87, .97]	1.0	.87	[.78, .95]	1.0

As highlighted in Fig 8, the Jensen-Shannon distances for the Δ_Sonja_ time lags are also low, ranging from 0.06 to 0.25. In addition, the density distributions of the RIP and EMA distance measures mostly overlap.

### Spatial measure

For the spatial dimension, we report the inhalation amplitude across dimensions and sentence types in Table 7 and show the correlation between the RIP signal and EMA distance measures in Table 8. While these are values averaged across speakers, speaker-specific values can be found in the OSF project.

**Table 7:** Speech inhalation amplitude. The parameter is expressed as standard deviations of the mean trajectory. Values are presented as the median with the IQRs in brackets by dimension and sentence type.

	sentence type			
dimension	short simple	short complex	long simple	long complex
RIP	2.67 (2.32 - 3.32)	2.9 (2.25 - 3.26)	2.58 (2.26 - 3.2)	2.49 (1.74 - 2.89)
D_chestmid,x_	2.9 (2.42 - 3.31)	2.66 (2.16 - 3.87)	2.51 (1.96 - 3.57)	2.43 (1.6 - 3.01)
D_chestmid,y_	2.69 (2.37 - 3.32)	2.75 (2.31 - 3.54)	2.54 (1.83 - 3.52)	2.62 (1.78 - 3.1)
D_midpoint,x_	2.64 (2.34 - 3.16)	2.71 (2.24 - 3.73)	2.65 (1.97 - 3.31)	2.53 (1.66 - 2.94)
D_midpoint,y_	2.64 (2.37 - 3.33)	2.82 (2.34 - 3.47)	2.6 (1.88 - 3.4)	2.61 (1.73 - 3.06)
D_sternum,x_	2.91 (2.37 - 3.4)	2.73 (2.09 - 3.34)	2.63 (2.03 - 3.43)	2.43 (1.68 - 3.11)
D_sternum,y_	3.02 (2.4 - 3.75)	3.05 (2.64 - 4.13)	2.89 (2.03 - 3.73)	2.7 (1.95 - 3.34)

**Table 8:** Correlation coefficients for inhalation amplitude. Means as well as the lower and upper boundaries of 95% of the posterior by EMA dimension and sentence type.

	sentence type											
	short simple			short complex			long simple			long complex		
dimension	$\hat{𝐩}$	95% HDI	Pr($\hat{𝐩}$ >0)	$\hat{𝐩}$	95% HDI	Pr($\hat{𝐩}$ >0)	$\hat{𝐩}$	95% HDI	Pr($\hat{𝐩}$ >0)	$\hat{𝐩}$	95% HDI	Pr($\hat{𝐩}$ >0)
D_chestmid,x_	.18	[-.17, .52]	.84	.44	[.13, .72]	.99	.47	[.18, .73]	1.0	.42	[.9, .71]	.99
D_chestmid,y_	.16	[-.18, .51]	.82	.41	[.9, .7]	.99	.5	[.23, .75]	1.0	.48	[.18, .75]	1.0
D_midpoint,x_	.18	[-.15, .53]	.85	.44	[.14, .73]	.99	.53	[.28, .78]	1.0	.55	[.3, .79]	1.0
D_midpoint,y_	.1	[-.26, .44]	.71	.39	[.8, .69]	.99	.52	[.25, .76]	1.0	.53	[.28, .78]	1.0
D_sternum,x_	.32	[-.1, .63]	.96	.71	[.51, .88]	1.0	.57	[.33, .8]	1.0	.55	[.29, .78]	1.0
D_sternum,y_	.1	[-.25, .46]	.7	.44	[.13, .72]	.99	.51	[.24, .76]	1.0	.58	[.33, .8]	1.0

When comparing amplitude values derived from RIP with those from EMA distance measures, again, the x-axis dimensions aligned most closely with RIP. Medians differed by less than 0.2 units on average, and their IQRs overlapped widely. For example, in short complex sentences, the inhalation amplitude was 2.9 [–0.87, 2.37] in RIP and 2.66 [–0.92, 2.49] in D_chestmid,x_, showing nearly identical magnitude and variability. Again, y-axis dimensions exhibited slightly higher medians and broader IQRs. Across all sentence types, amplitude values ranged from 2.4 to 3.0. This overall pattern can be inferred from Table 7: The inhalation amplitude is higher in short sentences than in long sentences. Within short sentences, the amplitude values are similar between simple and complex sentences, while within long sentences, the amplitude is often higher in simple sentences than in complex ones.

The posterior estimates for the inhalation amplitude in Table 8 show mostly moderate correlations between RIP signals and EMA distance measures. Similar to the inhalation duration, higher correlations were observed in short complex, long simple, and long complex sentences, whereas short simple sentences were the exception, showing only low correlation coefficients. In short simple sentences, the posterior estimates of the correlations for almost all EMA distance measures reveal low correlations, except for D_sternum,x_ where the posterior was $\hat{p}$ = .32 [−.10, .63]. In contrast, correlation coefficients extracted from short complex sentences ranged from $\hat{p}$ = .39 [.80, .69] in D_midpoint,y_ to $\hat{p}$ = .71 [.51, .88] in D_sternum,x_. In long simple sentences, values ranged from $\hat{p}$ = .47 [.18, .73] in D_chestmid,x_ to $\hat{p}$ = .57 [.33, .80] in D_sternum,x_. The range of correlation coefficients in long complex sentences ranged from $\hat{p}$ = .42 [.9, .71] in D_chestmid,x_ to $\hat{p}$ = .58 [.33, .80] in D_sternum,y_.

Jensen–Shannon distance for inhalation amplitude values range between 0.08 and 0.26 (see Fig 8). More noticeably differences in the EMA signal pattern from RIP are present in long simple and long complex sentences, showing values above 0.20, particularly in the long–simple and long–complex conditions.

### Dynamic trajectory comparison in RIP and EMA

For a dynamic analysis, we compared the entire trajectories between the signal type dimensions (EMA vs RIP) across entire sentence productions in time and space. By using cross-correlations, we investigated how similar the two signals were over time. Cross-correlation works by systematically shifting one signal relative to the other and computing their similarity at each possible time lag. Similarity is conveyed by the mean cross-correlation coefficients and their standard deviation by sentence type and the EMA distance measures compared to the RIP signal (Table 9). The cross-correlation coefficients represent the strength of the temporal alignment between the two signals, with values closer to 1 indicating highly similar trajectories.

**Table 9:** Cross-correlations between EMA and RIP by distinct EMA distance measures and sentence type.

	sentence type			
dimension	short simple	short complex	long simple	long complex
D_chestmid,x_	0.91 (0.84 – 0.95)	0.95 (0.89 – 0.97)	0.94 (0.89 – 0.96)	0.96 (0.90 – 0.97)
D_chestmid,y_	0.91 (0.77 – 0.96)	0.93 (0.88 – 0.97)	0.94 (0.88 – 0.96)	0.95 (0.90 – 0.97)
D_midpoint,x_	0.91 (0.84 – 0.96)	0.95 (0.91 – 0.98)	0.95 (0.91 – 0.98)	0.95 (0.91 – 0.98)
D_midpoint,y_	0.91 (0.80 – 0.95)	0.94 (0.88 – 0.96)	0.94 (0.88 – 0.97)	0.96 (0.90 – 0.97)
D_sternum,x_	0.91 (0.86 – 0.96)	0.95 (0.92 – 0.98)	0.96 (0.91 – 0.97)	0.95 (0.92 – 0.97)
D_sternum,y_	0.85 (0.67 – 0.94)	0.90 (0.84 – 0.96)	0.92 (0.86 – 0.96)	0.92 (0.87 – 0.96)

Overall, the cross-correlation coefficients are strongly positive, ranging from 0.85 to 0.96, indicating a high degree of similarity between the trajectories of the RIP signal and each EMA distance across sentence types. There are slight deviations related to sentence type, where the lowest coefficients were found for short simple sentences. There, the cross-correlation coefficients ranged from 0.85 for D_sternum,y_ to 0.91 for the remaining distances. Regarding the other sentence types, the cross-correlation coefficients were less variable. In short complex sentences, the cross-correlation coefficients ranged from 0.9 for D_sternum,y_ to 0.95 for D_chestmid,x_, D_midpoint,x_, and D_sternum,x_. Likewise, the D_sternum_ sensor marks also the range of the cross-correlation coefficients for long simple sentences, that is 0.92 in the y-dimension and 0.96 in the x-dimension. In long complex sentences, D_sternum,y_ marks also the lower end with 0.92. D_chestmid,y,_ D_midpoint,x_, and D_sternum,x_ had an identical median cross-correlation coefficients with 0.95. Similarly, D_chestmid,x_ and D_midpoint,y_ had the highest cross-correlation of 0.96.

**Fig 9:**
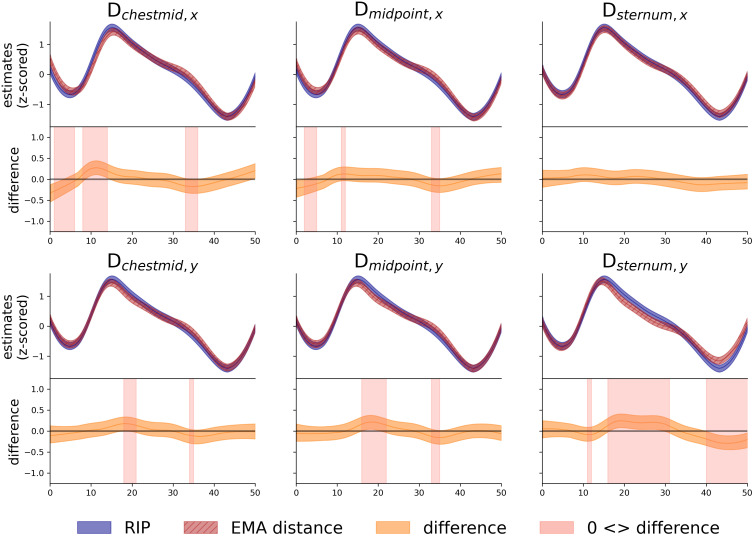
Trajectory analysis of short complex sentences. **Each panel consists of two plots and represents the comparison of RIP (blue) with one of the EMA distance measures.** The upper plots show 95% of the posterior estimates and the lower plots depict the difference between the RIP and the EMA distance. The time ranges where the differences are outside the 0 are highlighted by light red areas.

Fig 9 supports that there is very high consistency of the trajectories extracted from the EMA distances and the trajectories from the RIP signal. The figure displays the estimates and differences of the Bayesian-based trajectory analyses for short complex sentences. Within each panel, the upper plot displays the trajectory estimates while the lower plot shows the difference between RIP and the EMA distance in question. The blue trajectories display the trajectory estimates for the RIP signal, while each panel shows the trajectory estimates for the respective EMA distance in red. In the lower panels, the red shaded areas highlight time intervals where the difference between RIP and the EMA signal is large enough that it is unlikely to be due to chance (the solid horizontal line marks “no difference”). These shaded regions therefore show the moments where the two trajectories differ.

Besides very high consistency of the trajectories extracted from the EMA distances and the trajectories form the RIP signal, it can be observed that some EMA signals may show slight deviations from the RIP signals: Importantly, these deviations usually occur only during the inhalation or exhalation phases of the breathing cycle (and not at the starts or ends). For all dimensions, the differences emerge primarily during the middle of the inhalation phase or within the exhalation phase. Crucially, no notable differences appear at the inhalation onset or the inhalation offset/exhalation onset, indicating that the EMA trajectories are indeed comparable to important RIP landmarks. Minor discrepancies can be seen near the exhalation offset for D_sternum,y_, but, as illustrated in Fig 9, these deviations reflect small amplitude shifts rather than systematic changes in the overall shape of the breathing cycle. Potentially, these differences in amplitude may reflect differences in signal resolution, suggesting that residual lung volume at the end of an utterance may potentially be captured more accurately by EMA than by RIP. Similar results can be found in the OSF project for the remaining sentence types.

## Discussion & conclusion

In this work, we conducted a comparative analysis of speech breathing data collected via EMA and the more conventional RIP system, i.e., the Inductotrace (Ambulatory Monitoring, Inc.). Our results demonstrate a very high degree of correspondence between the two measurement techniques, supporting the feasibility of using EMA as a sole, integrated methodology for capturing the dynamics of speech articulation and speech breathing.

More specifically, the data show that horizontal EMA distance measures (front-back) closely replicate the temporal dynamics of the RIP signal, particularly regarding inhalation phase durations. In contrast, vertical movements (low-high) exhibit slightly lower medians and wider IQRs compared to RIP, indicating a bit more variability in the tracking of respiratory timing. The highest correlations between RIP and EMA were observed for D_chestmid,x_, as well as D_midpoint,x_, suggesting that the horizontal movement of the chest’s middle sensor as well as the calculated midpoint between the left and right chest sensor and the reference sensor provides the most accurate correspondence with RIP. The exhalation phase further confirms this pattern: horizontal EMA distances capture exhalatory timing with high correlation with the RIP, whereas vertical distances yield slightly longer and more variable medians, likely reflecting sensor sensitivity to abdominal or vertical thoracic motion. Again, D_chestmid,x_ and D_midpoint,x,_ show to be the most stable EMA distances measures when being compared to RIP. However, differences between horizontal and vertical EMA distances compared to RIP are comparatively small, and the results show strong overlap and high correlation of the measurements extracted from the EMA distances with the ones calculated from the RIP signal.

These findings are further supported by the temporal alignment between speech acoustics and speech breathing pattern, expressed by the variable Δ_Sonja_. The close correspondence between the onset of inhalation and the onset of speech acoustics demonstrates that both signals align consistently across acoustic and kinematic domains. In terms of spatial characteristics, the strong similarity, particularly between D_chestmid,x_ and RIP, indicates that horizontal EMA distance measures also provide a robust estimate of speech breathing amplitude.

The Jensen–Shannon distances likewise show a high degree of overlap between RIP and EMA dimensions. Exhalation duration seems to be the parameter for which EMA most closely approximates RIP, aligning with results on sustained vowel phonation, where exhalation phases were comparable across systems, while inhalation phases tend to be slightly shorter with EMA [44]. At the level of entire speech breathing trajectories, the movement contours obtained with EMA and RIP were highly similar, which is consistent with the present findings. Correlations also suggest that D_sternum,x_ can serve as an alternative sensor signal with minimal information loss. Additionally, speech breathing modulation driven by sentence length and linguistic load was reflected similarly across both systems and is consistent with previous findings; for example, inhalation durations were longer before long sentences than before short sentences [2]. However, unlike earlier results, our data might suggest a trend indicating that syntactic complexity may also influence inhalation timing. This potential effect requires more detailed analysis and statistical validation in future work, as the present paper was not aimed at investigating this aspect but rather at comparing RIP and EMA.

### Recommended set-up

To determine which EMA distance measure best corresponds to RIP, we tested several sensor positions and calculated multiple distance measures relative to the reference sensor. Overall, D_chestmid,x_ and D_midpoint,x_ provided the most comparable estimates to RIP, supporting their use as valid proxies for speech breathing research. These findings suggest that placing one sensor centrally on the rib cage and the reference one at the lowest cervical vertebra is well representing the RIP signal. However, out of simplicity and less intervening with the participants, we promote the set-up with one sensor on the sternum and one reference sensor on the lowest cervical vertebra): It is easy to apply across participants, as the sternum sensor can be placed quickly and reliably without requiring participants to remove clothing. Its easily accessible position ensures consistent sensor contact across different body types, and avoids interference with clothing. Despite its simplicity, this configuration still yields robust and stable speech breathing data and does not constrain participants’ comfort or natural movement. Crucially, only two sensors are required to track speech breathing kinematics, allowing all remaining EMA channels to be used for tracking articulatory movements. This enhances participant comfort and facilitates straightforward experimental preparation.

With regard to speech material, based on our experience using EMA for speech breathing analyses, we recommend avoiding very short sentences, as these are less reliably trackable. Some speakers may produce short sentences on residual lung volume without performing a preparatory inhalation, making them less suitable for analyzing speech breathing cycles. Longer sentences or continuous text reading that reliably trigger an inhalation are therefore preferable.

### Pros and Cons of using EMA

EMA offers several key advantages for simultaneously recording speech articulation and speech breathing. First, the ability to record articulatory and breathing kinematics, within a single, temporally synchronized system, enables detailed analyses of coordination between the two speech subsystems. Consequently, this approach facilitates the analysis of temporal coordination between acoustic, respiratory, and articulatory data using a single hardware solution. This represents a significant refinement over previous methodologies, such as that described by [24], by eliminating the need for data synchronization and substantially simplifying the post-processing workflow. Moreover, EMA provides high resolution 3D motion data that may surpass RIP in sensitivity and allows for advanced analytical approaches. Owing to this high spatial resolution, EMA may also be well-suited to investigating subtle residual breathing patterns.

However, some limitations must be considered. For studies focused exclusively on speech breathing, EMA may be unnecessarily complex, and traditional methods such as RIP or other plethysmographic techniques may offer simpler and more comfortable alternatives. Additionally, the comparatively high cost of EMA systems may pose a barrier for laboratories whose primary research emphasis is on speech breathing rather than articulation; in such cases, more affordable alternatives may be more appropriate depending on the study’s specific needs.

### Future applications

Our approach has potential for future applications across a wide range of linguistic and clinical research domains. Because speech and its breathing pattern are intrinsically linked in human sound production, the ability to capture both within a single, integrated EMA setup allows or comprehensive investigations of breathing–articulation coordination in continuous speech, dialogues, co-speech gestures, and even sign language, where breathing patterns associated with signing are relevant. Beyond core linguistic research, this methodology presents a particularly valuable asset for clinical applications, as both articulation and speech breathing function are often compromised in speech and voice disorders.

Future work should evaluate the reliability of EMA-based speech breathing measures in other postures, such as standing (with an additional sensor near the umbilicus), where others, for example accelerometers, have shown reduced accuracy [36]. Additionally, future research should evaluate the reliability of tracking abdominal movements using EMA sensors. This would facilitate the analysis of coordination between thoracic and abdominal contributions to speech breathing patterns across various postural conditions (e.g., sitting and standing). In addition, understanding speech breathing under challenged conditions, such as in aging populations, during loud speech, or under physical exertion [18], could offer new insights into compensatory strategies and functional impairments.

Using electromagnetic articulography can be expanded by utilizing the techniques’ capacity to capture precise 3D motion data. This opens up possibilities for more advanced metrics, including Euclidean distance-based analyses in 2D or 3D space, enabling a better understanding of both articulatory and breathing kinematics in speech. In sum, EMA represents a powerful tool for capturing speech kinematic data, i.e., in articulation and breathing, including the fine-grained temporal relationship between the two, without compromising measurement quality.

## Conclusion

The goal of this paper is to encourage other researchers to use EMA for the simultaneous assessment of speech articulation and speech breathing. Our approach opens new possibilities for investigating the coordination of these two critical speech subsystems. Such an integrated approach has the potential to yield richer datasets, facilitate the study of speech motor control integrating speech breathing, and enhance our understanding of the interdependence between articulation and breathing in speech production.
